# Glutaminolysis Mediated by MALT1 Protease Activity Facilitates PD-L1 Expression on ABC-DLBCL Cells and Contributes to Their Immune Evasion

**DOI:** 10.3389/fonc.2018.00632

**Published:** 2018-12-18

**Authors:** Xichun Xia, Wei Zhou, Chengbin Guo, Zhen Fu, Leqing Zhu, Peng Li, Yan Xu, Liangyan Zheng, Hua Zhang, Changliang Shan, Yunfei Gao

**Affiliations:** The First Affiliated Hospital, Biomedical Translational Research Institute and Guangdong Province Key Laboratory of Molecular Immunology and Antibody Engineering, Jinan University, Guangzhou, China

**Keywords:** diffuse large B-cell lymphoma, MALT1 protease activity, glutaminolysis, PD-L1, immune evasion

## Abstract

Previous studies have demonstrated that programmed death-1 ligand 1 (PD-L1) expressed in an aggressive activated B-cell (ABC)/non-germinal center B cell–like (GCB) subtype of diffuse large B-cell lymphoma (DLBCL) is associated with inhibition of the tumor-associated T cell response. However, the molecular mechanism underlying PD-L1 expression in ABC-DLBCL remains unclear. Here, we report that MALT1 protease activity is required for ABC-DLBCL cells to evade cytotoxity of Vγ9Vδ2 T lymphocytes by generating substantial PD-L1^+^ ABC-DLBCL cells. While, NF-κB was dispensable for the PD-L1 expression induced by MALT1 protease activity in ABC-DLBCL cells. Furthermore, we showed that GLS1 expression was profoundly reduced by MALT1 protease activity inhibition, which resulted in insufficiency of glutaminolysis-derived mitochondrial bioenergetics. Activation of the PD-L1 transcription factor STAT3, which was strongly suppressed by glutaminolysis blockade, was rescued in a TCA (tricarboxylic acid) cycle-dependent manner by glutamate addition. Collectively, MALT1 protease activity coupled with glutaminolysis-derived mitochondrial bioenergetics plays an essential role in PD-L1 expression on ABC-DLBCL cells under immunosurveillance stress. Thus, our research sheds light on a mechanism underlying PD-L1 expression and highlights a potential therapeutic target to vanquish immune evasion by ABC-DLBCL cells.

**Graphical Abstract:**
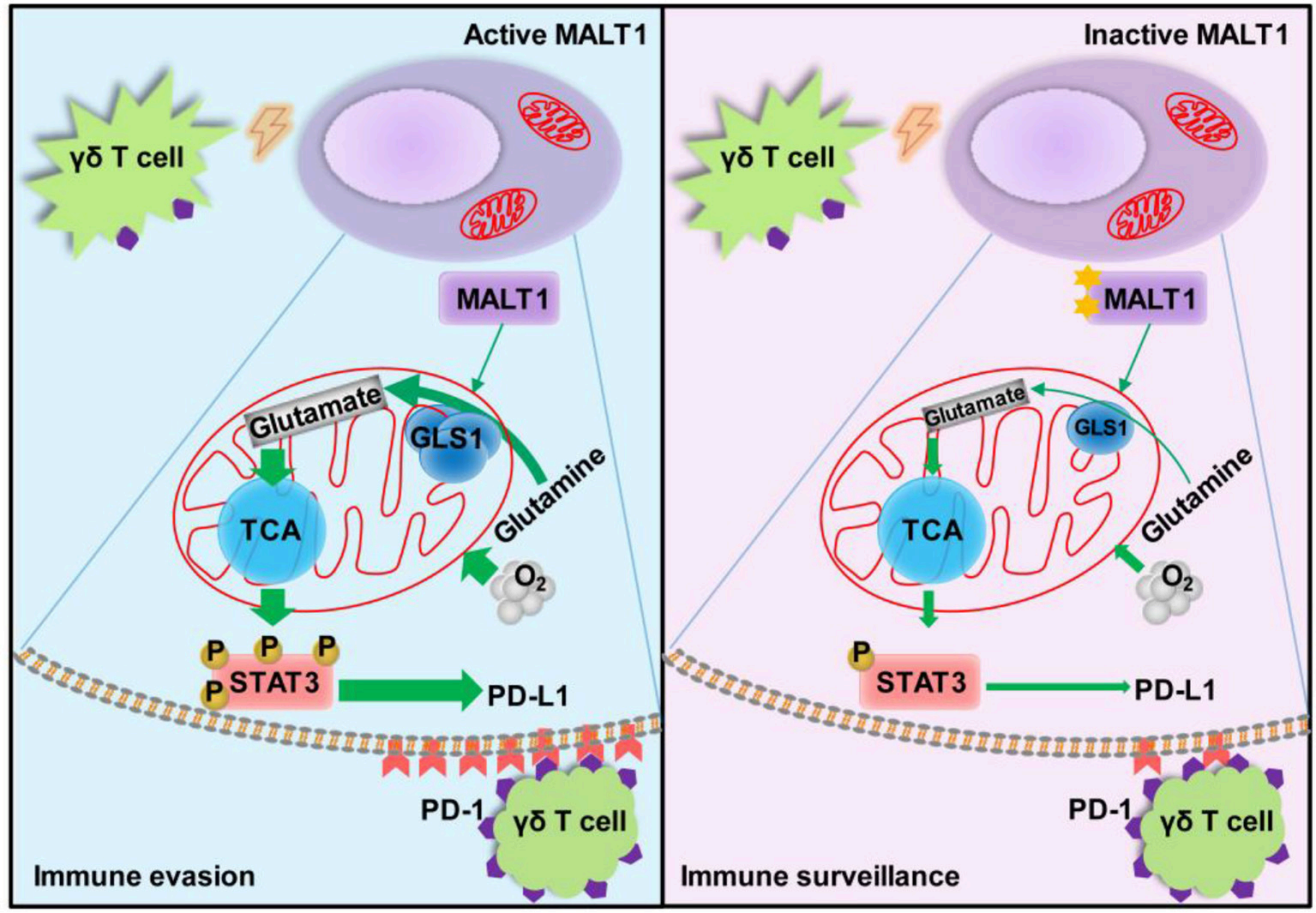
Under Vγ9Vδ2 T lymphocytes stress, the glutaminolysis intermediate glutamate enhances mitochondrial bioenergetics, resulting in STAT3 activation and PD-L1 expression in ABC-DLBCL cells. This process is controlled by MALT1 protease activity via up-regulating GLS1 expression. These results show a novel regulatory pathway that endows ABC-DLBCL cells with an immunosuppressive property. Hence, manipulation of these pathways could hold enormous potential for the development of effective immunotherapy for ABC-DLBCL.

## Introduction

Diffuse large B-cell lymphoma (DLBCL) comprises the largest subgroup of non-Hodgkin lymphoma (NHL), accounting for ~25% of all lymphoma cases (1). Gene expression profiling has allowed subclassification of DLBCL into at least two distinct molecular subtypes, including germinal center B cell–like (GCB) and activated B cell–like (ABC) DLBCL (2). The ABC subtype is aggressive, and patients with the ABC subtype have significantly shorter overall survival (OS) than those with the GCB subtype (3). Currently, R-CHOP (rituximab, cyclophosphamide, doxorubicin, vincristine, and prednisone) is the first-line treatment for DLBCL patients and has contributed to an overall improvement in patient survival (4). However, many patients with DLBCL are unresponsive to the treatment and generally have a poor prognosis, particularly patients with the ABC subtype (5, 6). This may be related to the antiapoptotic effects of NF-κB that counteract the action of cytotoxic chemotherapy, since NF-κB activity is usually high in this tumor type (7).

Tumor cells exploit the expression of programmed death-1 (PD-1) ligand 1 (PD-L1) to subvert T-cell-mediated immune-surveillance. In recent years, oncotherapy targeting the PD-1/PD-L1 immune-checkpoint has shown promising results in the treatment of various carcinoma types, such as melanoma, renal cancer, and lung cancer (8). A study on B-cell NHL demonstrated that PD-L1 expression was confined to a subset of DLBCL with activated B-cell features, which carries a poorer prognosis and frequently recurs after conventional chemoimmunotherapy (9). PD-L1-positive DLBCL was significantly associated with non-GCB type and the OS of patients with PD-L1-positive DLBCL was inferior to that of patients with PD-L1-negative DLBCL (10). Blockade of PD-1 can restore the anti-tumor functions of T-cells in DLBCL-derived cell lines *in vitro* (11). Together, this evidence suggests that GCB-DLBCL and the aggressive ABC/non-GCB subtype of DLBCL use distinct molecular mechanisms to regulate PD-L1 expression, which is preferentially used by the latter to escape recognition and killing by T cells.

The success of therapies that disrupt PD-L1-mediated tumor tolerance highlights the need to understand the molecular regulation of PD-L1 expression (12). Recently, many studies have focused on the mechanism underlying PD-L1 expression. Georgiou et al. found that translocations between *PD-L1* and the *IGH* locus led to PD-L1 overexpression in DLBCL, and this genetic alteration in the *PD-L1* locus is mainly associated with the non-GCB subtype of DLBCL (13). Further studies found that PD-L1 expression was regulated by kinase-cascade signaling pathways, transcription factors, and epigenetic factors. Both the PI3K/AKT and MAPK pathways are involved in controlling PD-L1 expression (14). Transcription factors, including regulatory elements responsive to IFN regulatory factor 1 (IRF1), NF-κB, hypoxia-inducible factor 1α (HIF1α), and STAT3, were found to bind to the PD-L1 gene promoter (15–17). Furthermore, recent reports provide a possible link between metabolic reprogramming and PD-L1 expression (18, 19). Oversupply of the glycolytic intermediate pyruvate to mitochondria enhances PD-L1 expression by fostering oxidative phosphorylation and TCA cycle activity in macrophages (19). However, our knowledge of PD-L1 expression regulation in DLBCL and the biological functions of the regulation is limited.

Mucosa-associated lymphoid tissue lymphoma translocation gene 1 (MALT1), originally identified in B-cell lymphoma, is a Cys-dependent, Arg-specific protease (20). After antigenic stimulation, MALT1 forms part of the CARMA1-BCL10-MALT1 (CBM) complex and catalyzes protease activity that cleaves inhibitors of the NF-κB signaling pathway, such as TNFAIP3/A20, BCL10 protein, CYLD, and RELB (21). This indirectly activates NF-κB signaling. Constitutive NF-κB activation mediated by MALT1 protease activity is observed in the ABC-DLBCL subtype and is linked to its pathogenesis. Inhibition of MALT1 protease activity or expression of a catalytically inactive form of MALT1 dramatically reduced the viability of cell lines derived from ABC-DLBCL, while cell lines derived from other B cell lymphoma types, such as GCB-DLBCL, Burkitt's lymphoma, and marginal zone lymphoma, were not affected (22). Recently, small molecule inhibitors of MALT1 were developed that efficiently suppressed ABC-DLBCL in xenograft experiments and patient samples *ex vivo* (23). These evidences indicate that MALT1 protease activity is required for the survival of ABC-DLBCL but not GCB-DLBCL. Although PD-L1 expression is regulated by NF-κB in cancer cells (24), it remains an open question whether MALT1 protease activity regulates PD-L1 expression and the PD-L1-mediated immune-evasion in ABC-DLBCL.

In this study, we report that MALT1 protease activity is essential for PD-L1 expression in ABC-DLBCL cells under Vγ9Vδ2 T lymphocytes stress. We found that MALT1 protease activity supported glutaminolysis by up-regulating expression of the enzyme GLS1, resulting in higher glutamate production. Subsequently, glutamate enters the TCA cycle to enhance STAT3 activation and PD-L1 expression. Thus, MALT1 protease activity supports glutaminolysis and contributes to ABC-DLBCL cell immune evasion.

## Materials and Methods

### Cell Culture and Reagents

The human DLBCL cell lines BJAB, U2932, OCI-Ly3 were obtained from DSMZ, SUDHL-4, and SUDHL-6 were obtained from American Type Culture Collection (ATCC; Manassas, VA, USA). All cell lines were cultured in RPMI 1640 medium supplemented with 20% FBS and 100 U/ml penicillin/streptomycin (Gibco). OCI-Ly10 was purchased from Cobioer Biosciences Co., LTD (Nanjing, China) and cultured in IMDM with 20% FBS, 100 U/ml penicillin/streptomycin (Gibco) and 50 μM β-mercaptoethanol (Sigma-Aldrich). All cell lines were cultured at 37°C in a humidified atmosphere of 5% CO_2_.

z-VRPR-fmk (Enzo Life Sciences) was dissolved in ddH_2_O at a concentration of 50 μM throughout all experiments. MI-2, BPTES, QNZ, CPI-613 (Selleck), and PMA/Iono (Sigma-Aldrich) were reconstituted in DMSO (final DMSO concentration 0.1%) and their final concentrations were 1 μM, 2 μM, 5 μM, 100 μM, and 50/500 ng/ml, respectively.

### Generation of Vγ9Vδ2 T Lymphocytes

PBMCs from fresh blood samples of healthy adult donors were isolated using density gradient centrifugation with Ficoll-Paque (GE Healthcare). To generate Vγ9Vδ2 T lymphocytes, freshly isolated PBMCs were cultured in RPMI 1640 medium supplemented with 10% heat-inactivated FBS, 100 U/ml penicillin/streptomycin (Gibco) and 50 μM β-mercaptoethanol at a density of 3 X 10^6^ cells/mL in a 24-well plate. At the onset of the culture, 30 μM zoledronic acid (Sigma-Aldrich) and 400 U/mL IL-2 (PeproTech, Inc.) were added. Three days later, the medium was replaced with maintenance medium containing 10 μg/mL IL-2, and the maintenance medium was changed every 2–3 days. The cells were selectively expanded after 10–14 days. This study was carried out in accordance with the recommendations of local ethics guidelines, the Ethics committee of Jinan University with written informed consent from all subjects. All subjects gave written informed consent in accordance with the Declaration of Helsinki. The protocol was approved by the Ethics committee of Jinan University. Experimental procedures have been carried out following the standard biosecurity and the institutional safety procedures.

### Cytotoxicity Assay

The cytotoxic potential of expanded Vγ9Vδ2 T lymphocytes against DLBCL cells was assayed using propidium iodide (PI, Sangon Biotech Shanghai Co., Ltd.) staining. In brief, CFSE-labeled Vγ9Vδ2 T lymphocytes were co-incubated with DLBCL cells at different effector:target (E:T) ratios of Vγ9Vδ2 T lymphocytes to DLBCL cells (0:1, 5:1, 10:1, and 20:1) at 37°C for 6 h. After cells were washed with PBS, PI solution was added to the co-culture and incubated with the cells for 5 min. Subsequently, the proportion of PI^+^CFSE^−^ cells was quantified using flow cytometry. In some experiments, DLBCL cells were pretreated with z-VRPR-fmk, BPTES or QNZ for 12 h prior to co-culture with Vγ9Vδ2 T lymphocytes. For blocking assays, purified anti-human CD274 antibody (10 μg/ml; 29E.2A3) or isotype control antibody (10 μg/ml; MG2b-57; BioLegend) were included at the time of effector/target co-culture.

### Real-Time RT-PCR

Total RNA was extracted with an RNA Simple Total RNA kit (Tiangen Biotech Beijing Co., Ltd.). Synthesis of cDNA was performed with DNA-free RNA samples (Qiagen) using reverse transcription with abPrimeScript™ RT reagent kit (Takara) according to the manufacturer's protocol. Real-time PCR was performed using SYBR Green qPCR Master Mix (Bimake) on a LC480 Lightcycler system (Roche). RNA was normalized to β-actin mRNA. The sequences of primers were as follows (5′ to 3′): (forward) TTC GGT CCA GTT GCC TTCT and (reverse) GGT GAG TGG CTG TCT GTG TG for IL-6, (forward) TGG GGG AGA ACC TGA AGA and (reverse) ATG GCT TTG TAG ATG CCT TTC for IL-10, (forward) GTG AGT CGG ATC GCA GCTT and (reverse) TCG GCT GCT GCA TTG TTC for Bal-xl, (forward) CAT GTA CGT TGC TAT CCA GGC and (reverse) CTC CTT AA TGTC ACG CAC GAT for β-actin.

### Flow Cytometry

To measure the proportion and differential subsets of Vγ9Vδ2 T lymphocytes, we performed surface staining with FITC-conjugated anti-CD3 (Tianjin Sungene Biotech Co., Ltd.), PerCP-conjugated anti-TCR Vδ2, Pacific Blue-conjugated anti-CD27 and APC-conjugated anti-CD45RA (BioLegend) antibodies. To evaluate the functional markers of Vγ9Vδ2 T lymphocytes, cells were restimulated with 50 ng/ml PMA and 500 ng/ml ionomycin for 6 h, permeabilized using reagents from a BD Cytofix/Cytoperm Kit and stained with PE-Cy7-conjugated anti-NKG2D, APC-conjugated anti-IFN-γ (BD Biosciences), PE-conjugated anti-Perforin and Pacific Blue-conjugated anti-Granzyme B (BioLegend) antibodies. GolgiStop (BD Biosciences) was added for the last 4 h of culture. To evaluate intracellular protein, DLBCL cells were sorted from the co-culture, fixed, permeabilized using BD Phosflow™ (BD Biosciences) and stained with Alexa Fluor® 647-conjugated anti-STAT3 Phospho (Tyr705) (Biolegend). To measure PD-L1^+^ or PD-1^+^ cells, Vγ9Vδ2 T lymphocytes were co-incubated with DLBCL cells for 6 h. We then performed surface staining with BV421-conjugated anti-PD-L1 (Biolegend) or Pacific Blue-conjugated anti-PD-1 (Biolegend) antibodies. For the cell survival assay, an Annexin V/PI apoptosis kit (Bimake) was used. The data were analyzed using FlowJo software (Tree Star, lnc).

### Western Blotting

Cell pellets were collected after washing with PBS. Total cellular protein was extracted using lysis buffer (20 mM Tris-HCl (pH 7.4), 150 mM NaCl, 1 mM EDTA, 1 mM EGTA, 1% Triton X-100, 2.5 mM sodium pyrophosphate, 1 mM β-glycerophosphate, 1 mM sodium orthovanadate, 1 μg/ml leupeptin, 1 mM phenylmethylsulfonyl fluoride), and the protein concentration was determined with a BCA Kit (Pierce). After boiling in SDS-PAGE loading buffer, 20–40 μg of protein in total cell lysates was fractionated via SDS-PAGE and transferred to PVDF membranes (Millipore). Blots were blocked in 5% BSA (Sigma-Aldrich) and incubated with the appropriate antibodies. The primary antibodies anti-Bcl-10, anti-RelB, anti-p65/Phospho-p65, anti-AKT/Phospho-AKT, anti-ERK/Phospho-ERK, anti-STAT3/Phospho-STAT3 (Cell Signaling), anti-Bcl-xl (Proteintech), anti-GLS1, anti-VDAC1 (Sangon Biotech Shanghai Co., Ltd.), and anti-β-actin (Tianjin Sungene Biotech Co., Ltd.) were used. Goat-anti-mouse IgG/HRP and goat-anti-rabbit IgG/HRP (Cell Signaling) were the secondary antibodies used. The data were acquired using a ChemiDoc™ XRS+ System (Bio-Rad) with enhanced chemiluminescence HRP substrate (Millipore).

### Seahorse

OCR and ECAR were assessed using a Seahorse XF-96 extracellular flux analyzer (Seahorse Bioscience). DLBCL cells were pooled, carefully counted and plated (1 × 10^5^ cells/well) in assay medium onto Seahorse cell plates coated with Cell-Tak (Corning, Inc.). For OCR, assay medium was supplied with XF Base Medium with 1 mM pyruvate, 2 mM glutamine, and 10 mM glucose (Sigma-Aldrich). Baseline OCR was defined as the OCR readings before 1 μM oligomycin (Seahorse Bioscience) injection. SRC was defined as the difference between baseline OCR and maximal OCR after carbonyl cyanide-4-(trifluoromethoxy) phenylhydrazone (FCCP, 0.5 μM, Seahorse Bioscience) injection to uncouple oxidative phosphorylation and electron transport. For ECAR, assay medium was supplied with XF Base Medium with 2 mM glutamine. Glycolysis was defined as the ECAR reached by a given cell after the addition of saturating amounts of glucose. Glycolytic reserve was defined as the difference in ECAR between the glucose and 1 μM oligomycin injections.

### Confocal Microscopy

Treated DLBCL cell lines were washed with RPMI 1640 medium without phenol red and stained with MitoTracker Green (ThermoFisher) for 30 min at 37°C. After washing, cells were resuspended with PBS and dropped in a glass slides, cover-slipped with a fluoroshield mounting medium with DAPI (Abcam). Images were collected on a Leica TCS SP8 confocal microscopy.

#### Mitochondria Isolation and Mitochondrial ATP Assay

Mitochondria isolation was performed using Qproteum mitochondria isolation kit as manufacturer' protocol (Qiagen, USA). The ATP assay kit was from Beyotime and the assay was performed according to manufacturer's protocol. After centrifugation to remove cell debris, the supernatant was added to the substrate solution. The luminescence was recorded in an Illuminometer with an integration time of 10 s per well.

### Analysis of [U-^13^C]-Glucose Metabolites and Glutamate Assay

U2932 cells were grown in [U-^13^C]-glucose medium and were treated with z-VRPR-fmk or not for 12 h. After collection, 1 mL of chloroform and 1 mL of methanol/H_2_O solvent (v/v = 1:1) were successively added into a tube of cells. The suspension was vortexed for 30 s and incubated at 4°C for 2 h. The mixture was processed using 7 cycles of ultrasonication for 2 min and incubated at −40°C for 2 min before centrifugation at 16, 000 g and 4°C for 15 min. In total, 400 μL of supernatant was transferred into a glass vial containing 10 μL of internal standards (0.05 mg/mL 13C6-15N-L-isoleucine), and dried under a gentle nitrogen stream. The dry residues were reconstituted in 30 μL of 15 mg/mL methoxylamine hydrochloride in anhydrous pyridine. The resulting mixture was vortex-mixed vigorously for 30 s and incubated at 37°C for 90 min. Then, 30 μL of MTBSTFA (with 1% BDMCS) was added to the mixture and derivatized at 55°C for 60 min. Metabolomic instrumental analysis was performed on an Agilent 7890A gas chromatography system coupled to an Agilent 5975C inert MSD system (Agilent Technologies Inc.). An HP-5ms fused-silica capillary column (30 m × 0.25 mm × 0.25 μm; Agilent J&W Scientific, Folsom, CA) was utilized to separate the derivatives. Helium (> 99.999%) was used as a carrier gas at a constant flow rate of 1 mL/min through the column. The injection volume was 1 μL (splitless mode), and the solvent delay time was 5.5 min. The initial oven temperature was held at 100°C for 2 min, ramped to 140°C at a rate of 10°C/min, to 260°C at a rate of 5°C/min, to 320°C at a rate of 10°C/min, and finally held at 320°C for 8 min. The temperatures of the injector, transfer line, and electron impact ion source were set to 250°C, 250°C, and 230°C, respectively. The electron energy was 70 eV, and data were collected in full scan mode (m/z 50–600). At least three separate injections were measured per sample, and the percent enrichment of ^13^C-labeled metabolites in the total metabolite pool was calculated for each metabolite.

Glutamate assays were performed according to the manufacturer's protocols (Sigma-Aldrich).

### Statistical Analysis

Two-tailed, unpaired or paired Student's *t*-test was used to compare differences between treated and control groups using GraphPad Prism 6.0 software. Differences with *p*-values < 0.05 were considered statistically significant: ^*^p < 0.05; ^**^p < 0.01; ^***^p < 0.001.

## Results

### MALT1 Protease Activity Is Required for ABC-DLBCL Cell Escape From the Cytotoxicity of Human Vγ9Vδ2 T Lymphocytes

**Figure 1:**
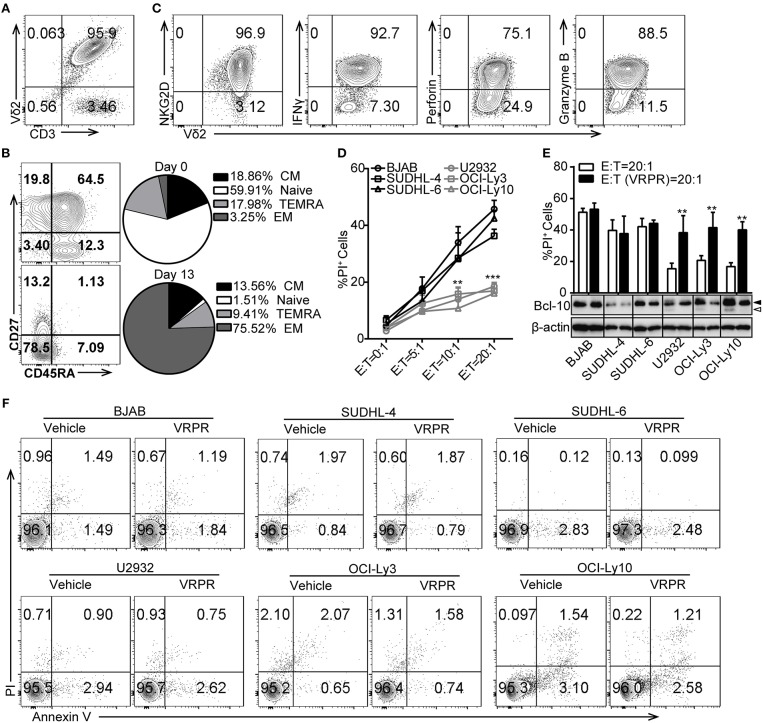
MALT1 protease activity is required for ABC-DLBCL cell escape from the cytotoxicity of human Vγ9Vδ2 T lymphocytes. **(A)** The purity of Vγ9Vδ2 T lymphocytes expanded *in vitro* on day 13. **(B)** Differentiation subsets of Vγ9Vδ2 T lymphocytes on day 0 and day 13. Vγ9Vδ2 T lymphocyte subsets were subdivided according to CD27 and CD45RA expression: naïve (CD45RA^+^CD27^+^), CM (CD45RA^−^CD27^+^), EM (CD45RA^−^CD27^−^), and TEMRA (CD45RA^+^CD27^−^) (*n* = 5). **(C)** The percentage of NKG2D^+^, IFN-γ^+^, perforin^+^ and Granzyme B^+^ cells within the Vγ9Vδ2 T lymphocyte population. **(D)** Cytotoxicity of Vγ9Vδ2 T lymphocytes toward DLBCL cells at the indicated effector to target ratios (E:T) (*n* = 5). **(E)** Cytotoxicity of Vγ9Vδ2 T lymphocytes toward DLBCL cells pretreated with vehicle or z-VRPR-fmk (VRPR) for 12 h (*n* = 5). Western blot to detect Bcl-10 cleavage products; Filled arrow, full length Bcl-10; open arrow, Bcl-10 cleavage product; β-actin was used as the loading control. **(F)** Survival of DLBCL cells pretreated with vehicle or VRPR for 12 h followed by apoptosis analysis via flow cytometry. The data are representative of three independent experiments. Data are shown as the means ± SD, ^**^p < 0.01; ^***^p < 0.001.

To investigate whether ABC-DLBCL and GCB-DLBCL cells exert different immune-evasion effects on T lymphocytes, we expanded Vγ9Vδ2 T lymphocytes by culturing human peripheral blood mononuclear cells (PBMCs) from healthy adults and co-cultured them with three ABC-DLBCL cell lines (U2932, OCI-Ly3, and OCI-Ly10) and three GCB-DLBCL cell lines (BJAB, SUDHL-4, and SUDHL-6) *in vitro*. Expanded Vγ9Vδ2 T lymphocytes typically accounted for more than 95% of the cultured PBMCs (Figure 1A) and were mainly effector memory (EM, CD45RA^−^CD27^−^) cells, which displayed efficient cytotoxic activity and cytokine production (Figure 1B). Flow cytometric analysis confirmed that the expanded Vγ9Vδ2 T lymphocytes expressed the innate receptor NKG2D and produced IFN-γ, the cytolytic protein perforin, and granzyme B (Figure 1C). As expected, ABC-DLBCL cells showed fewer PI^+^ populations than GCB-DLBCL cells when selected by Vγ9Vδ2 T lymphocytes at the various E/T ratios tested (Figure 1D), suggesting that ABC-DLBCL cells were more inclined to subvert the cytotoxicity of Vγ9Vδ2 T lymphocytes. To further illustrate whether MALT1 protease activity is involved in the tolerance of ABC-DLBCL cells to Vγ9Vδ2 T lymphocytes, we pretreated DLBCL cell lines with z-VRPR-fmk, a cell-permeable and irreversible MALT1 inhibitor. z-VRPR-fmk treatment efficiently blocked the proteolytic activity of MALT1, as indicated by the absence of the cleaved-BCL10 fragment (Figure 1E). Interestingly, all three z-VRPR-fmk pretreated ABC-DLBCL cell lines doubled their cell death in response to Vγ9Vδ2 T lymphocytes (Figure 1E). In stark contrast, z-VRPR-fmk did not have a significant effect on any of the three GCB-DLBCL cell lines tested (Figure 1E). Moreover, z-VRPR-fmk alone had no impact on the survival of these DLBCL cells (Figure 1F). These results indicated that MALT1 protease activity has an essential role in assisting ABC-DLBCL cell escape from the cytotoxicity of Vγ9Vδ2 T lymphocytes.

### PD-L1 Is Involved in the MALT1 Protease Activity-mediated Immunosuppressive Property of ABC-DLBCL Cells

**Figure 2:**
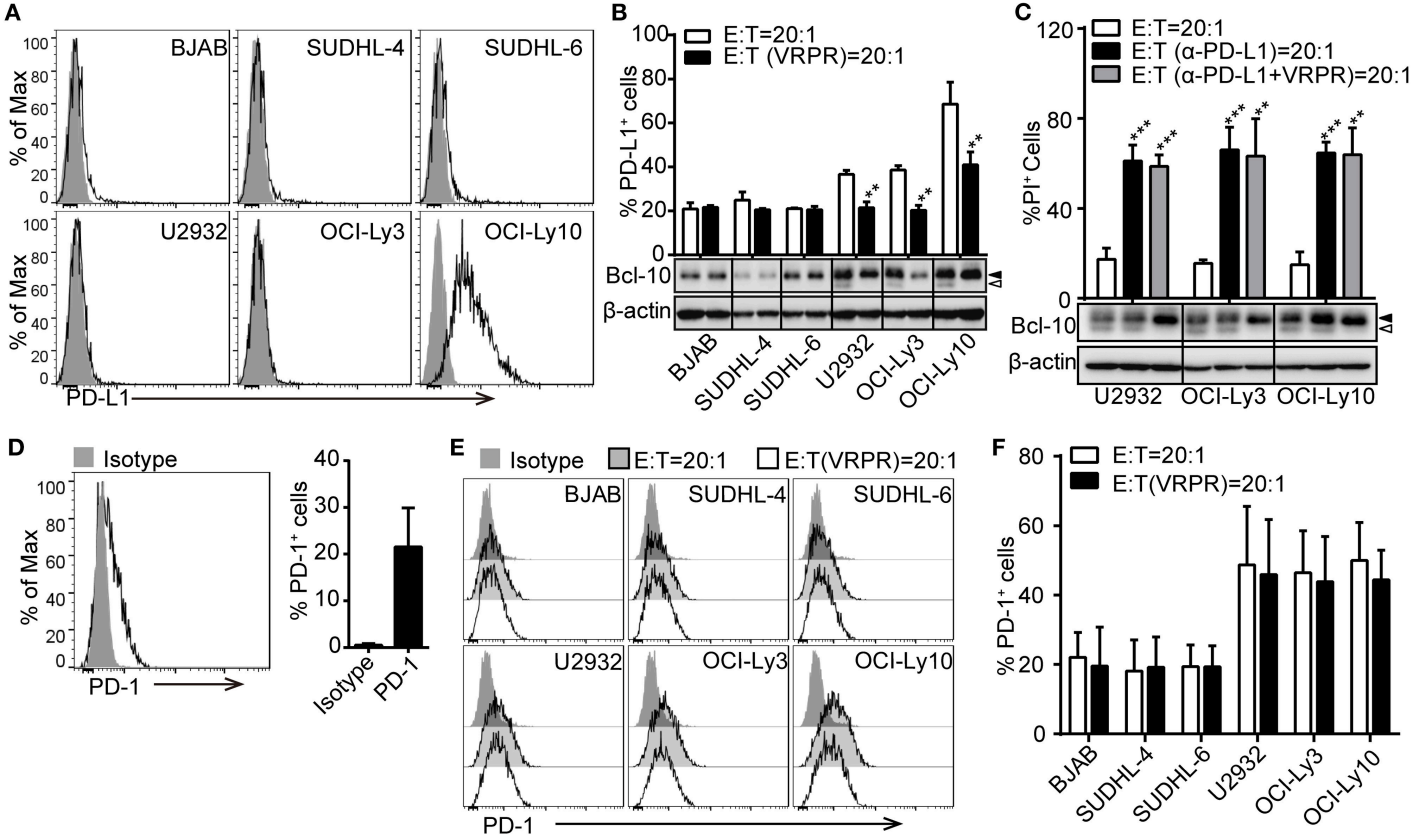
PD-L1 is involved in the MALT1 protease activity-mediated immunosuppressive property of ABC-DLBCL cells. **(A)** Flow cytometric analysis of DLBCL cell lines for PD-L1. One representative experiment of three is depicted. The black curve in each histogram show staining by specific mAbs, and isotype control results are shown in gray. **(B)** DLBCL cells were pretreated with vehicle or VRPR for 12 h prior to exposure to CFSE-labeled Vγ9Vδ2 T lymphocytes for 6 h. Proportions of PD-L1^+^ DLBCL cells within DLBCL cells were detected by flow cytometry (*n* = 5). **(C)** Cytotoxicity of Vγ9Vδ2 T lymphocytes toward ABC-DLBCL cells pretreated with vehicle, anti-PD-L1 antibody, or anti-PD-L1 antibody + VRPR (α-PD-L1+ VRPR) for 12 h (*n* = 5). Western blot to detect Bcl-10 cleavage products; β-actin was used as the loading control. **(D)** Flow cytometric analysis of Vγ9Vδ2 T lymphocytes for PD-1 (*n* = 10). The black curve in each histogram show staining by specific mAbs, and isotype control results are shown in gray. **(E)** CFSE-labeled DLBCL cells were pretreated with vehicle or VRPR for 12 h prior to exposure to Vγ9Vδ2 T lymphocytes for 6 h. Expression of PD-1 on Vγ9Vδ2 T lymphocytes was detected by flow cytometry (*n* = 4). **(F)** Proportion of PD-1^+^ Vγ9Vδ2 T lymphocytes from the graph in **(E)** are shown (*n* = 4). Data are shown as the means ± SD, **p < 0.01; ***p < 0.001.

To confirm that PD-L1 plays a key role in ABC-DLBCL cell resistance to the cytotoxicity of Vγ9Vδ2 T lymphocytes, we screened a panel of DLBCL cell lines for PD-L1 expression using flow cytometry. Surprisingly, only the OCI-Ly10 cell line showed high levels of PD-L1 expression. The others, including three GCB-DLBCL cell lines (BJAB, SUDHL-4 and SUDHL-6) and two ACB-DLBCL cell lines (U2932 and OCI-Ly3), expressed little PD-L1 (Figure 2A). Considering that PD-L1 expression is immunologically active by suppressing the activation of tumor-associated T cells (9, 25), we checked the kinetics of PD-L1 expression on these DLBCL cell lines in response to Vγ9Vδ2 T lymphocytes. We observed that the proportion of PD-L1^+^ cells increased in the DLBCL cell lines, suggesting that Vγ9Vδ2 T lymphocyte stress might promote PD-L1 expression (Figure 2B). Interestingly, the proportion of PD-L1^+^ ABC-DLBCL cells was generally higher than that of PD-L1^+^ GCB-DLBCL cells. Treatment with z-VRPR-fmk significantly decreased the proportion of PD-L1^+^ ABC-DLBCL cells (Figure 2B). These results revealed that MALT1 protease activity is essential for PD-L1 expression on ABC-DLBCL cells. Importantly, blocking PD-L1 with anti-PD-L1 antibodies effectively restored the cytotoxicity of Vγ9Vδ2 T lymphocytes (Figure 2C). In addition, administration of anti-PD-L1 antibody induced no further increase in cytotoxicity when combined with z-VRPR-fmk, suggesting that z-VRPR-fmk enhances Vγ9Vδ2 T lymphocyte cytotoxicity toward ABC-DLBCL cells mainly by inhibiting PD-L1 expression (Figure 2C). On the other hand, we found that an average of ~20% of the expanded Vγ9Vδ2 T lymphocytes expressed PD-1 in the basal state (Figure 2D). The proportion remained nearly the same when Vγ9Vδ2 T lymphocytes were incubated with GCB-DLBCL cells, but markedly increased when they were mixed with ABC-DLBCL cells (Figures 2E,F). However, the PD-1 expression remained unchanged in Vγ9Vδ2 T lymphocytes incubated with GCB-DLBCL or ABC-DLBCL cells pretreated with z-VRPR-fmk (Figures 2E,F). Together, these data support the idea that z-VRPR-fmk selectively decreases the generation of PD-L1^+^ ABC-DLBCL cells in response to Vγ9Vδ2 T lymphocytes, thereby inhibiting the immunosuppressive property of ABC-DLBCL cells.

### NF-κB Is Dispensable for PD-L1^+^ ABC-DLBCL Cells Generation Mediated by MALT1 Protease Activity

**Figure 3:**
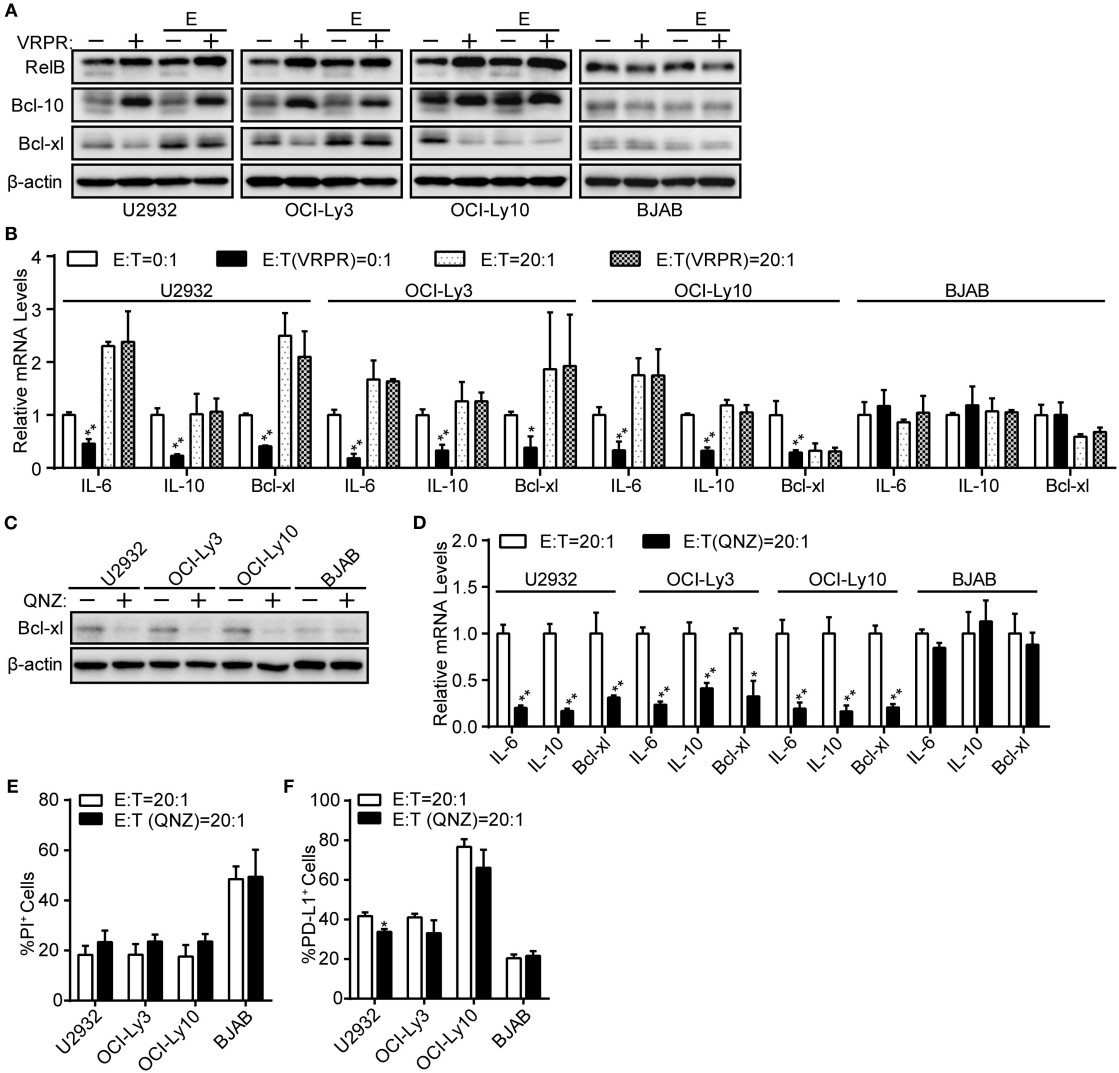
NF-κB is dispensable for PD-L1^+^ ABC-DLBCL cells generation mediated by MALT1 protease activity. **(A)** ABC-DLBCL cells and BJAB cells were pretreated with VRPR (+) or not (-) for 12 h prior to exposure to CFSE-labeled Vγ9Vδ2 T lymphocytes (E) or not for 6 h. Bcl-xl, cleavage products of RelB or Bcl-10 were detected in sorted DLBCL cells by western blotting. β-Actin was used as the loading control. One representative experiment of three is depicted. **(B)** qRT-PCR analysis of relative IL-6, IL-10, and Bcl-xl mRNA levels in ABC-DLBCL cells and BJAB cells from **(A)**. **(C)** ABC-DLBCL and BJAB cells were pretreated with QNZ (+) or not (–) for 12 h before being exposed to CFSE-labeled Vγ9Vδ2 T lymphocytes for 6 h. Bcl-xl was detected in sorted DLBCL cells by western blotting. β-Actin was used as the loading control. One representative experiment of three is depicted. **(D)** qRT-PCR analysis of relative IL-6, IL-10, and Bcl-xl mRNA levels in ABC-DLBCL cells and BJAB cells from **(C)**. **(E)** Cytotoxicity of Vγ9Vδ2 T lymphocytes toward ABC-DLBCL cells or BJAB cells from **(C)** (*n* = 3). **(F)** Proportions of PD-L1^+^ ABC-DLBCL cells or BJAB cells from **(C)** (*n* = 3). Data are shown as the means ± SD, *p < 0.05; **p < 0.01.

NF-κB, a common transcription factor downstream of the CBM complex, has been shown to regulate PD-L1 expression in tumor cells (24, 26). Thus, we assessed whether NF-κB is indispensable for the PD-L1 expression on ABC-DLBCL cells mediated by MALT1 protease activity. We found that NF-κB transcription activity was significantly inhibited in ABC-DLBCL cells based on the diminished expression of NF-κB target genes, including Bcl-xl protein expression (Figure 3A) and the mRNA levels of Bcl-xl, IL-6, and IL-10 (Figure 3B), in response to z-VRPR-fmk treatment. However, the abolished NF-κB transcription activity induced by z-VRPR-fmk recovered in the presence of Vγ9Vδ2 T lymphocytes (Figures 3A,B). We excluded the possibility that z-VRPR-fmk lost its NF-κB inhibition activity by including BJAB cells as a negative control; in these cells, NF-κB target genes remained constant under these conditions. Since the proportion of PD-L1^+^ ABC-DLBCL cells was profoundly decreased by z-VRPR-fmk (Figure 2B), NF-κB might not be involved in the PD-L1^+^ ABC-DLBCL cell generation under the stress of Vγ9Vδ2 T lymphocytes. To confirm the role of NF-κB in generating PD-L1^+^ ABC-DLBCL cells, we directly blocked NF-κB transcription using QNZ. Distinct from z-VRPR-fmk, QNZ completely blocked NF-κB transcription under Vγ9Vδ2 T lymphocyte stress, as shown by the extreme decreases in Bcl-xl protein expression (Figure 3C) and the Bcl-xl, IL-6, and IL-10 mRNA levels (Figure 3D). Importantly, QNZ-induced blockage of NF-κB transcription activity only slightly increased cell death in ABC-DLBCL cells (Figure 3E) and slightly decreased PD-L1^+^ABC-DLBCL cell generation (Figure 3F). Taken together, NF-κB is dispensable for the generation of PD-L1^+^ ABC-DLBCL cells mediated by MALT1 protease activity under Vγ9Vδ2 T lymphocyte stress.

### MALT1 Protease Activity Endowed ABC-DLBCL Cells With Latent Mitochondrial Bioenergetics Ability

**Figure 4:**
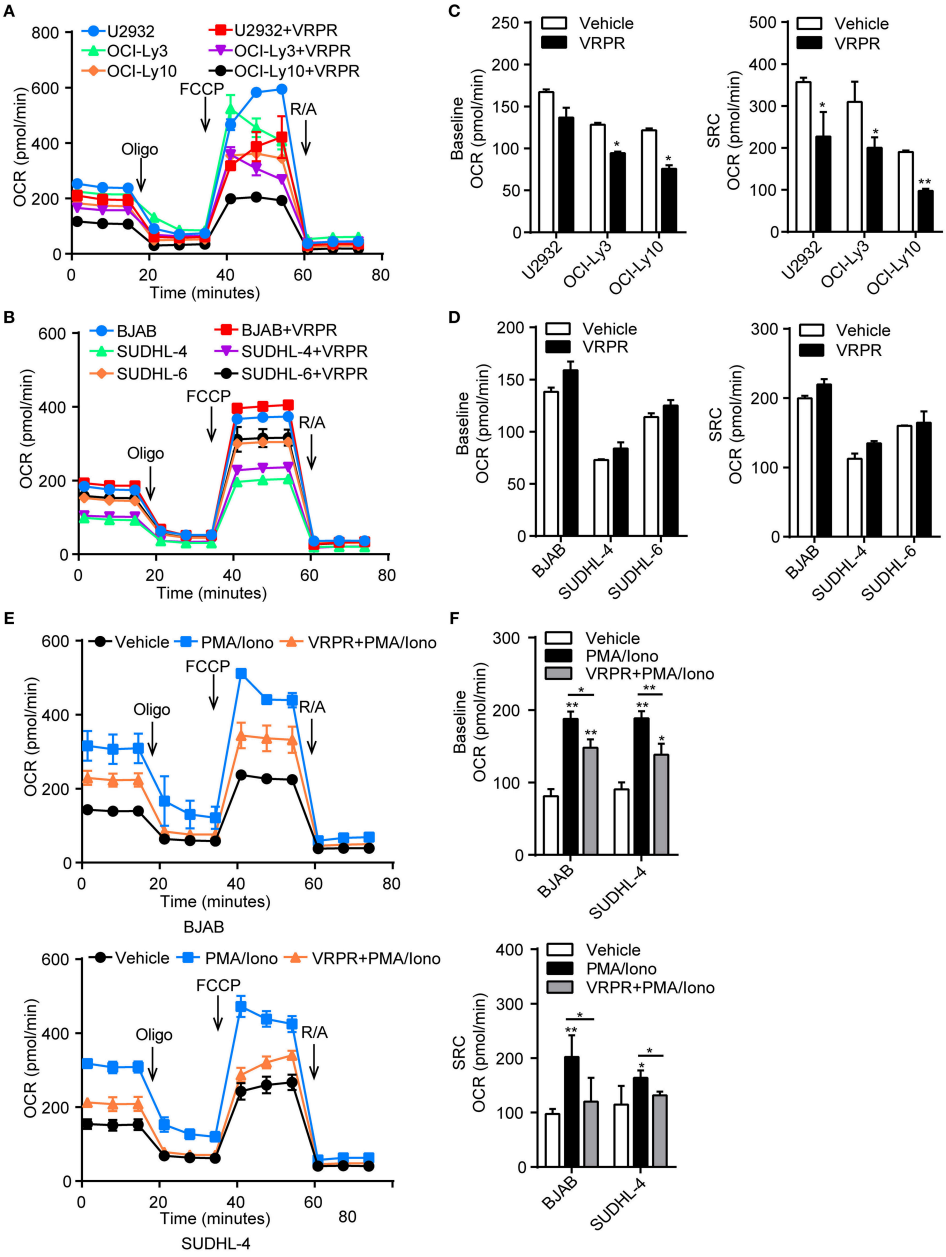
MALT1 protease activity endowed ABC-DLBCL cells with latent mitochondrial bioenergetics ability. Mitochondrial respiration profiles in ABC-DLBCL cells **(A)** and GCB-DLBCL cells **(C)** treated with vehicle or VRPR. **(B)** Baseline OCR and mitochondrial spare respiratory capacity (SRC) of the graphs in **(A)** were calculated. **(D)** Baseline OCR and SRC of the graphs in **(C)** were calculated. **(E)** Mitochondrial respiration profiles in GCB-DLBCL cells (BJAB and SUDHL-4 cells) that were either left untreated or stimulated for 12 h with PMA/Iono (P/I); zVRPR-fmk was added 12 h before stimulation where indicated. **(F)** Baseline OCR and SRC of the graphs in **(E)** were calculated. All the graphs represent as the mean ± SD of three independent experiments. *p < 0.05; **p < 0.01.

A possible link was recently reported between metabolic alterations and PD-L1 expression (18, 19). Hence, we proposed that MALT1 protease activity might be involved in regulating cell metabolic reprogramming to support PD-L1^+^ ABC-DLBCL cell generation. To test our hypothesis, we examined the bioenergetic profiles of z-VRPR-fmk- or vehicle-treated DLBCL cells, after confirming z-VRPR-fmk efficiently blocked the proteolytic activity of MALT1 (Supplementary Figure 1A). We found that the basal oxygen consumption rate (OCR) was slightly lower in ABC-DLBCL cells primed with z-VRPR-fmk, while mitochondrial spare respiratory capacity (SRC) was substantially decreased, as revealed through the difference between basal OCR and maximal OCR after FCCP treatment (Figures 4A,B). This absent mitochondrial SRC suggested that mitochondria in these cells were operating close to their bioenergetics limit under stressful conditions, which was further confirmed by decreased ATP-coupled OCR and mitochondrial ATP levels (Supplementary Figure 1B). However, mitochondria biogenesis was not influenced by VRPR, which was indicated by unchanged VDAC1 protein levels and intensity of MitoTracker Green staining (Supplementary Figures 1C,D). For GCB-DLBCL cells, these were not influenced by treatment with z-VRPR-fmk (Figures 4C,D; Supplementary Figures 1B–D). Furthermore, we found that GCB-DLBCL cells (BJAB and SUDHL-4 cells) with activated MALT1 protease activity presented higher basal OCR and mitochondrial SRC than the control group or the z-VRPR-fmk combined with PMA/Iono group (Supplementary Figure 2A; Figures 4E,F). We also measured the changes in extracellular acidification rate (ECAR), a typical readout of cellular glycolytic activity, in these DLBCL cells by administering z-VRPR-fmk (Supplementary Figures 2B,C). The data were analyzed and reported as the rate of glycolysis under basal conditions and glycolytic reserve calculated as the difference between the maximal glycolytic capacity and the basal glycolysis, which revealed unchanged after z-VRPR-fmk treatment (Supplementary Figures 2D,E). All the above data showed that MALT1 protease activity endowed ABC-DLBCL cells with a latent ability for mitochondrial bioenergetics, which might be fully applied in response to Vγ9Vδ2 T lymphocytes.

### MALT1 Protease Activity-supported Mitochondrial Bioenergetics Is Dependent on Glutaminolysis

**Figure 5:**
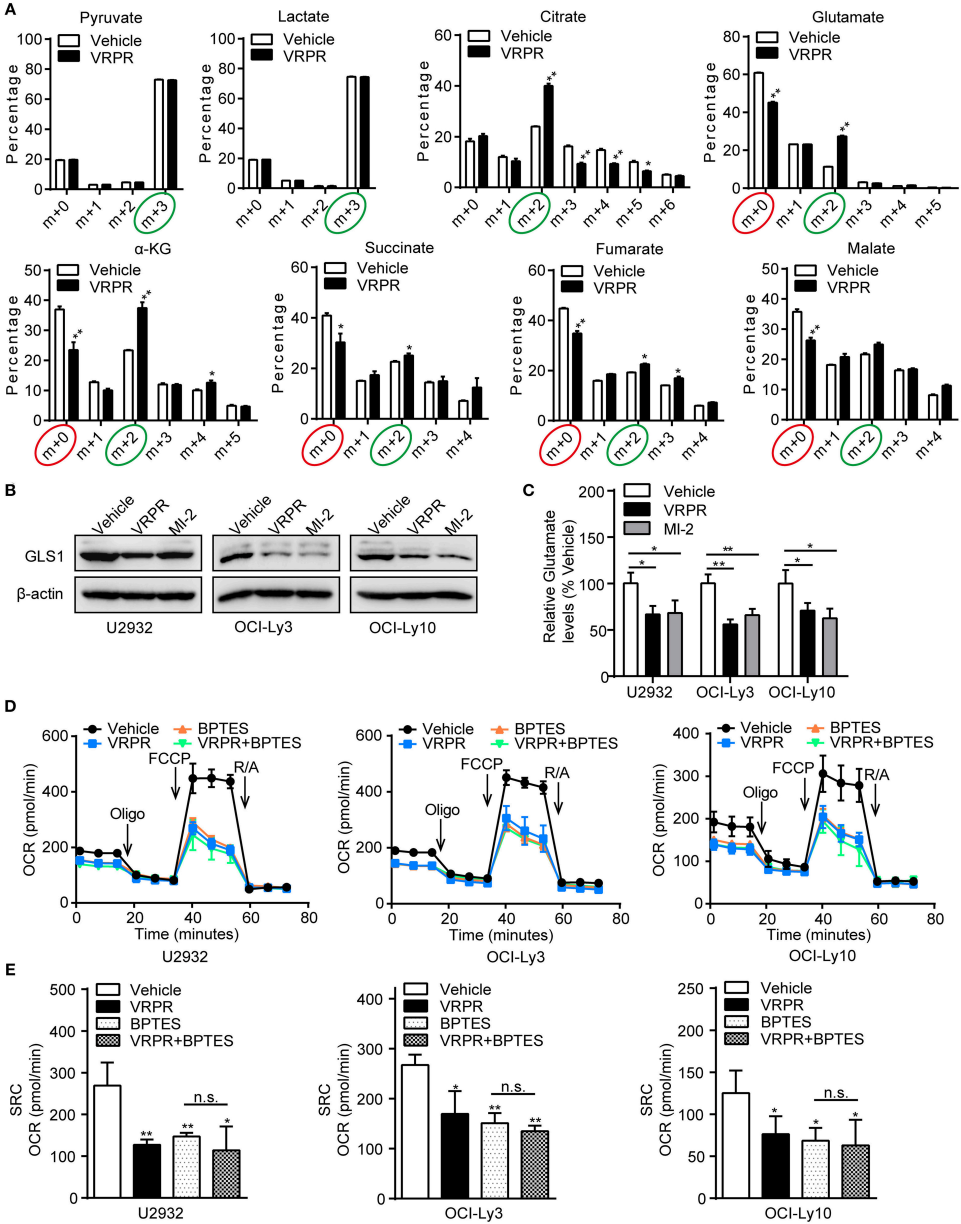
MALT1 protease activity-supported mitochondrial bioenergetics is dependent on glutaminolysis. **(A)** ^13^C isotopomer analysis of uniformly labeled glucose (U^13^C_6_-glucose) in U2932 cells treated with z-VRPR-fmk or not for 12 h. The isotopolog distributions were determined by GC-MS. The incorporation of ^13^C atoms from ^13^C_6_-Glc into pyruvate, lactate, citrate, a-ketoglutarate (a-KG), succinate, fumarate, and malate are denoted as m+n, where n is the number of ^13^C atoms. **(B)** Western blot to detect GLS1 in ABC-DLBCL cells pretreated with vehicle, VRPR or MI-2 for 12 h. β-Actin blotting served as the loading control. One representative experiment of three is depicted. **(C)** Relative glutamate levels in ABC-DLBCL cells from **(B)**. **(D)** Profiles of mitochondrial respiration in ABC-DLBCL cells pretreated with vehicle, VRPR, BPTES, or VRPR+BPTES for 12 h. OCR was assayed after consecutive injections of Oligo (1 μM), FCCP (0.5 μM), rotenone (1 μM), and antimycin (1 μM). **(E)** OCR of the graphs in **(D)** were calculated. All the graphs represent as the mean ± SD of three independent experiments. *p < 0.05; **p < 0.01.

To understand how MALT1 protease activity supports mitochondrial bioenergetics in ABC-DLBCL cells, we analyzed the metabolic consequences in the U2932 cell line, whose MALT1 protease activity was profoundly blocked by z-VRPR-fmk (Supplementary Figure 3A). We grew these cells in uniformly labeled [U-^13^C]-glucose medium. As the results showed, there was no change in the level of the m+3 isotopolog of lactate (^13^C_3_-lactate) derived from [U-^13^C]-glucose, which indicated an unchanged ECAR of ABC-DLBCL cells primed with z-VRPR-fmk (Figure 5A). In addition, z-VRPR-fmk did not impact pyruvate derived from [U-^13^C]-glucose (m+3 forms). However, glucose-derived TCA cycle intermediates, such as the doubly ^13^C-labeled isotopolog of citrate, a-ketoglutarate, succinate, fumarate, and malate (m+2 forms, circled green), were increased (Figure 5A). Notably, a large fraction of these TCA metabolites (m+0 forms, circled red) were not derived from the [U-^13^C]-glucose (Figure 5A), suggesting that an alternative source supported mitochondrial bioenergetics via TCA cycling. Meanwhile, these TCA metabolites (m+0 forms, circled red) decreased under MALT1 protease activity inhibition conditions (Figure 5A). These data suggest that glutamate from glutaminolysis likely entered the TCA cycle, which was attenuated by MALT1 protease activity inhibition. Subsequently, we found that the levels of GLS1 protein (Figure 5B) and intracellular glutamate (Figure 5C) were down-regulated in ABC-DLBCL cells without MALT1 protease activity but up-regulated in GCB-DLBCL cells with MALT1 protease activity (Supplementary Figures 3B,C). These data indicate that MALT1 protease activity promoted glutaminolysis by enhancing GLS1 expression, producing substantial glutamate that entered the TCA cycle to support mitochondrial bioenergetics. Next, we determined whether enhanced mitochondrial SRC was attributed to glutaminolysis-mediated mitochondrial bioenergetics. We exposed ABC-DLBCL cells to the GLS1 inhibitor BPTES, which blocks glutaminolysis by inhibiting the chemical conversion of glutamine to glutamate (Supplementary Figure 3D). Treatment with either BPTES or z-VRPR-fmk impaired mitochondrial SRC in ABC-DLBCL cells (Figures 5D,E). Administration of BPTES showed no additional decline in mitochondrial SRC when combined with z-VRPR-fmk (Figures 5D,E), suggesting that the enhancement effect of MALT1 on mitochondrial SRC was dependent on glutaminolysis in ABC-DLBCL cells. Together, these data established that MALT1 protease activity up-regulated GLS1 expression, thereby promoting glutaminolysis-mediated mitochondrial bioenergetics in ABC-DLBCL cells.

### Glutaminolysis Enhanced PD-L1^+^ ABC-DLBCL Cell Generation to Subvert the Cytotoxicity of Human Vγ9Vδ2 T lymphocytes

**Figure 6:**
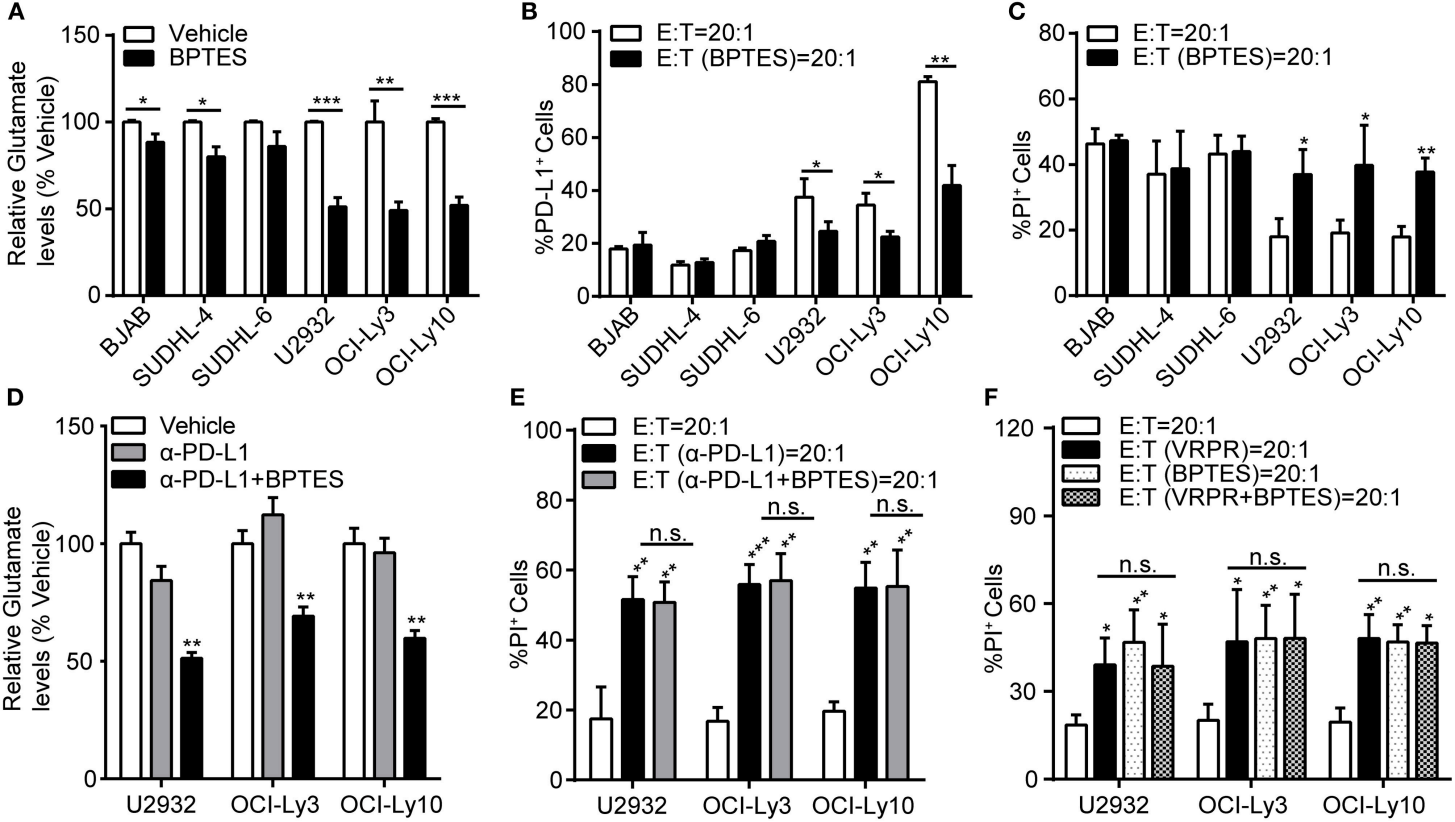
Glutaminolysis enhanced PD-L1^+^ ABC-DLBCL cell generation to subvert the cytotoxicity of human Vγ9Vδ2 T lymphocytes. **(A)** Relative glutamate levels in DLBCL cells pretreated with vehicle or BPTES for 12 h. **(B)** DLBCL cells were pretreated with vehicle or BPTES for 12 h before being exposed to CFSE-labeled Vγ9Vδ2 T lymphocytes for 6 h. Proportions of PD-L1^+^ DLBCL cells were detected (*n* = 5). **(C)** Cytotoxicity of Vγ9Vδ2 T lymphocytes toward DLBCL cells from **(B)** (*n* = 5). **(D)** Relative glutamate levels in DLBCL cells pretreated with vehicle, anti-PD-L1 antibody, or anti-PD-L1 antibody + BPTES (α-PD-L1+B) for 12 h. **(E)** Cytotoxicity of Vγ9Vδ2 T lymphocytes toward ABC-DLBCL cells from **(D)** (*n* = 5). **(F)** Cytotoxicity of Vγ9Vδ2 T lymphocytes toward ABC-DLBCL cells pretreated with vehicle, z-VRPR-fmk, BPTES, or z-VRPR-fmk + BPTES for 12 h (*n* = 5). Data are shown as the means ± SD, *p < 0.05; **p < 0.01; ***p < 0.001.

To explore whether glutaminolysis enhanced the generation of PD-L1^+^ ABC-DLBCL cells, we exposed DLBCL cells to BPTES and co-cultured them with Vγ9Vδ2 T lymphocytes. After treatment with BPTES, glutaminolysis was effectively blocked, as indicated by the decreased intracellular glutamate levels (Figure 6A), while survival of these DLBCL cells was not influenced before exposure to Vγ9Vδ2 T lymphocytes (Supplementary Figure 4). Compared with that of GCB-DLBCL cells, PD-L1^+^ cell generation was significantly depressed (Figure 6B) and ABC-DLBCL cell death was promoted (Figure 6C) by BPTES exposure. BPTES administration did not further improve the cytotoxicity of Vγ9Vδ2 T lymphocytes combined with anti-PD-L1 antibodies (Figures 6D,E), suggesting that the attenuation effect of BPTES on immune-suppressive function was mediated by PD-L1 expression in ABC-DLBCL cells. To determine the extent to which the anti-cytotoxicity effects of MALT1 protease activity in ABC-DLBCL cells are glutaminolysis-dependent, we pretreated ABC-DLBCL cells with z-VRPR-fmk and BPTES separately or in combination followed by co-culture with Vγ9Vδ2 T lymphocytes. The combination of both inhibitors did not increase the cell death rate compared with the effect of either inhibitor alone (Figure 6F), which suggested that glutaminolysis mediated the immunosuppressive effects of MALT1 protease activity on ABC-DLBCL cells.

### Mitochondrial Bioenergetics Mediated by Glutaminolysis Supports STAT3 Activation

**Figure 7:**
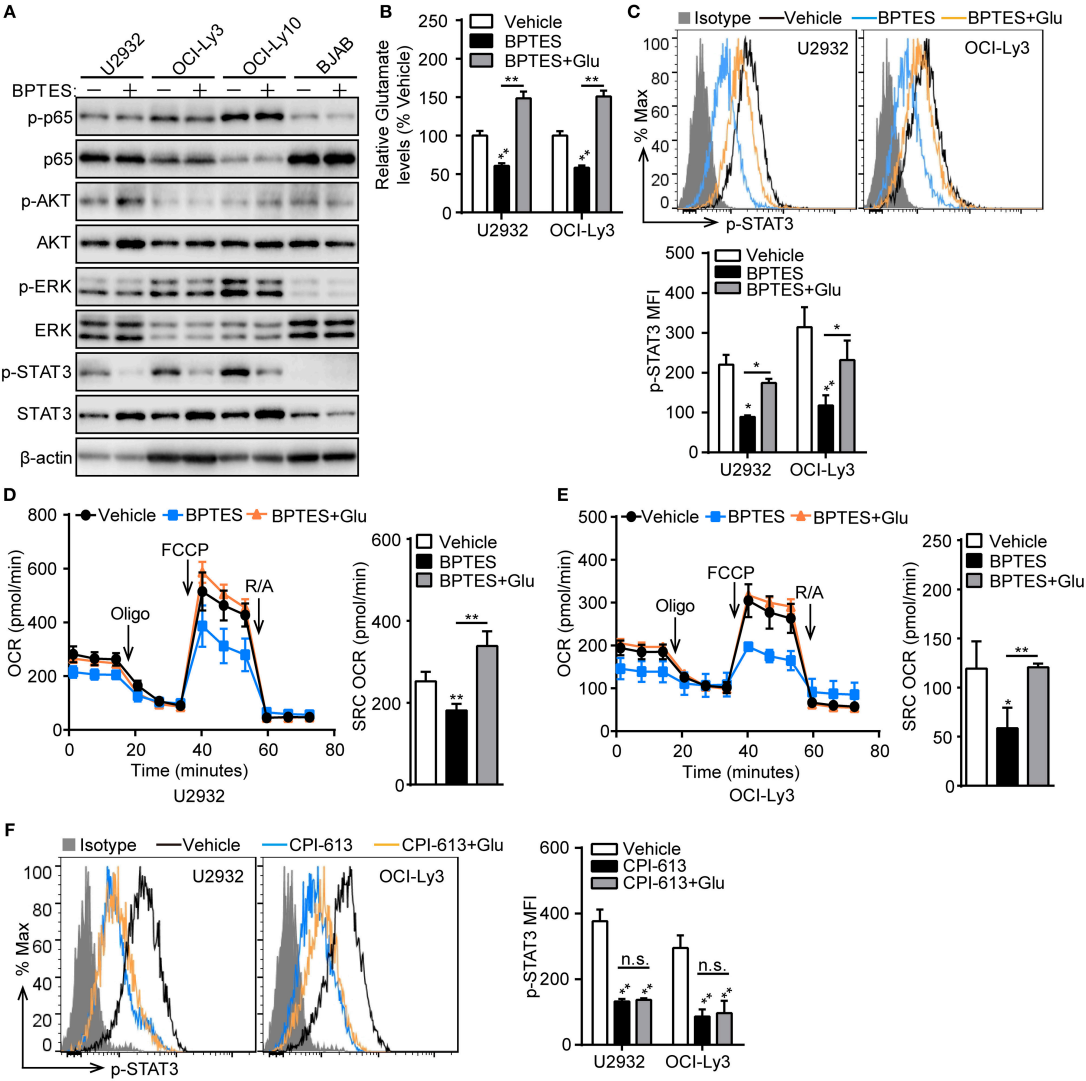
Mitochondrial bioenergetics mediated by glutaminolysis supports STAT3 activation. **(A)** ABC-DLBCL cells and BJAB cells were treated with BPTES (+) or not (−) for 12 h before being exposed to CFSE-labeled Vγ9Vδ2 T lymphocytes for 6 h. Western blot to detect changes in the indicated proteins in sorted DLBCL cells. **(B)** Relative glutamate levels of U2932 and OCI-Ly3 cells treated with vehicle, BPTES or BPTES + Glutamate (Glu) for 12 h. One representative experiment of three is depicted. **(C)** U2932 and OCI-Ly3 cells from **(B)** were exposed to CFSE-labeled Vγ9Vδ2 T lymphocytes for 6 h. Changes of p-STAT3 levels were detected in sorted U2932 and OCI-Ly3 cells by flow cytometry, and the MFI of p-STAT3 was calculated (*n* = 3). Mitochondrial respiration profiles in U2932 **(D)** and OCI-Ly3 cells **(E)** from **(B)** (*n* = 3). **(F)** U2932 and OCI-Ly3 cells were treated with vehicle, CPI-613 or CPI-613+Glutamate (Glu) for 12 h prior to exposure to CFSE-labeled Vγ9Vδ2 T lymphocytes for 6 h. Changes of p-STAT3 levels were detected in sorted U2932 and OCI-Ly3 cells by flow cytometry, and the MFI of p-STAT3 was calculated (*n* = 3). Data are shown as the means ± SD, *p < 0.05; **p < 0.01.

To understand how glutaminolysis promotes PD-L1^+^ ABC-DLBCL cell generation, we blocked glutaminolysis in ABC-DLBCL cells using BPTES and then co-cultured them with Vγ9Vδ2 T lymphocytes. After sorting DLBCL cells, we measured the changes in total and phosphorylated p65, AKT, ERK, and STAT3 protein levels. As shown in Figure 7A, STAT3 phosphorylation was almost completely inhibited by treatment with BPTES in ABC-DLBCL cells, indicating its activation was inhibited by blocking glutaminolysis. In contrast, p65, AKT and ERK phosphorylation was not impacted, suggesting that STAT3 is the major regulator of PD-L1. Re-supplementation of glutamate completely recovered the reduced levels of intracellular glutamate and phospho-STAT3 caused by BPTES treatment (Figures 7B,C), indicating that glutamate or glutamate-mediated mitochondrial bioenergetics plays an important role in maintaining phospho-STAT3. To further confirm that glutamate-mediated mitochondrial bioenergetics maintains phospho-STAT3 or glutamate, we detected the mitochondrial respiration profiles of ABC-DLBCL cells after glutamate re-supplementation in the presence of BPTES. ABC-DLBCL cells showed a lower mitochondrial SRC in the presence of BPTES, which recovered to levels as high as those observed in the original state when glutamate was resupplied, indicating that glutamate entered the TCA cycle to support mitochondrial bioenergetics (Figures 7D,E). Moreover, glutamate re-supplementation did not recover the phospho-STAT3 levels to those observed in the original state in cells treated with CPI-613, which functions as a TCA cycle blocker (Figure 7F). These data illustrate that glutaminolysis-mediated mitochondrial bioenergetics helped to sustain phospho-STAT3, a PD-L1 transcription factor, which finally led to generation of PD-L1^+^ ABC-DLBCL cells under Vγ9Vδ2 T lymphocyte stress.

## Discussion

PD-L1 expression is exploited by various tumor cells, including gastric cancer, hepatocellular carcinoma, renal cell carcinoma, esophageal cancer, pancreatic cancer, and ovarian cancer cells, to subvert T-cell-mediated immunosurveillance (27). Recently, evidences have shown that PD-L1 is also predominately expressed in the aggressive ABC/non-GCB subtype of DLBCL (13, 28). However, the molecular mechanism by which PD-L1 expression is regulated in ABC-DLBCL remains unknown. In this study, we report for the first time that MALT1 protease activity coupled to glutaminolysis contributes to PD-L1 expression on ABC-DLBCL cells, leading to immunosuppression against Vγ9Vδ2 T lymphocytes. This study reveals a novel mechanism of PD-L1 regulation and indicates that glutaminolysis restriction may support novel immunotherapy approaches for ABC-DLBCL.

Vγ9Vδ2 T lymphocytes are involved in tumor-immune surveillance, notably against carcinomas and hematologic malignancies (29). In contrast with αβ T lymphocytes, Vγ9Vδ2 T lymphocytes mediate potent antitumor effects in a HLA-unrestricted manner (30). In addition, they can be expanded routinely *in vitro*. More importantly, the expression of the ligand PD-L1 on tumor cells hampers the functional antitumor response of Vγ9Vδ2 T lymphocytes (31). These factors suggest that Vγ9Vδ2 T lymphocytes are a convenient and reasonable choice for studying immune evasion mediated by the PD-1/PD-L1 axis *in vitro*. GCB-DLBCL and ABC-DLBCL show distinct PD-L1 expression, and thus, first, we examined the immune evasion ability of ABC-DLBCL and GCB-DLBCL cells against Vγ9Vδ2 T lymphocytes. We observed that ABC-DLBCL cells were more inclined to subvert the cytotoxicity of Vγ9Vδ2 T lymphocytes than GCB-DLBCL cells. However, this immune evasion ability was significantly attenuated by inhibition of MALT1 protease activity. The proportion of PD-L1^+^ ABC-DLBCL cells was obviously reduced under Vγ9Vδ2 T lymphocyte stress prior to inactivation of MALT1 protease activity, while that of PD-L1^+^ GBC-DLBCL cells was much less reduced. In addition, PD-Ll blockage reduced the immune evasion function of ABC-DLBCL cells, but no further attenuation was seen by inhibiting MALT1 protease activity. These data revealed that PD-L1 expression on ABC-DLBCL cells causes immune evasion that is mediated by MALT1 protease activity. More interestingly, we noticed that the DLBCL cell lines used in our study are PD-L1 negative, except for the OCI-Ly10 cell line. Only when exposed to Vγ9Vδ2 T lymphocytes did these DLBCL cells express PD-L1. PD-L1 up-regulation on tumor cells might be a consequence of pro-inflammatory cytokines produced by tumor infiltrating immune cells. For example, IFN-γ produced by inflammatory cells acts as a potent PD-L1 up-regulator (15). Another study noted that PD-L1 expressed on tumor cells is immunologically active in suppressing tumor-associated T cell activation (9). Accordingly, the emergence of PD-L1^+^ DLBCL cells was likely induced by certain pro-inflammatory cytokines secreted by Vγ9Vδ2 T lymphocytes in our experimental system. Overall, we found that MALT1 protease activity is essential for PD-L1^+^ ABC-DLBCL generation under the immune pressure of Vγ9Vδ2 T lymphocytes.

Furthermore, we detected the scale of PD-1^+^ Vγ9Vδ2 T lymphocyte generation, which accounted for an average of ~20% of the lymphocytes on day 13 during *in vitro* culture. Our observations of the pattern of PD-1 expression by Vγ9Vδ2 T lymphocytes are consistent with those of other reports (32). Following antigenic stimulation, Vγ9Vδ2 T lymphocytes express high levels of PD-1 (29, 32, 33). Consequently, the proportion of PD-1^+^ Vγ9Vδ2 T lymphocytes significantly increased in the presence of ABC-DLBCL cells regardless of whether they were pretreated with or without z-VRPR-fmk. Apart from that, cytokines, including IL-2, IL-6, IL-7, IL-15, IL-21, IFN-α, and TNF-α, are also thought to induce PD-1 expression in activated T lymphocytes (34, 35). ABC-DLBCL cells ordinarily produce some of these cytokines (36, 37), which might be another reason for the increased generation of PD-1^+^ Vγ9Vδ2 T lymphocytes. Persuasively, we found that IL-6 mRNA levels were identical in ABC-DLBCL cells pretreated with either z-VRPR-fmk or vehicle, and these levels increased after exposure to Vγ9Vδ2 T lymphocytes. This hints that IL-6 may be one of the inducers of PD-1 expression on Vγ9Vδ2 T lymphocytes in our experimental system. Altogether, inducible PD-L1 and PD-1 provide favorable conditions for ABC-DLBCL cells to exert immune-suppressive activity. Inhibition of MALT1 protease activity potentially provides an approach for reversing increases in the proportion of PD-L1^+^ ABC-DLBCL cells, thereby weakening their immune-evasion property, although it does not influence the PD-1^+^ Vγ9Vδ2 T lymphocyte number. Therefore, MALT1 protease activity inhibition depressed the immunosuppressive property of ABC-DLBCL cells by attenuating the probability of PD-L1/PD-1 interaction.

MALT1 protease activity plays an essential role in activation of NF-κB, which functions as a transcription factor of PD-L1 in tumor cells (24). We originally speculated that NF-κB is indispensable for the ability of MALT1 protease activity to mediate PD-L1^+^ ABC-DLBCL cell generation. Unexpectedly, in the absence of NF-κB transcription activity, the proportion of PD-L1^+^ ABC-DLBCL cells was obviously decreased via MALT1 protease activity inhibition under Vγ9Vδ2 T lymphocyte stress. This suggested that another pathway, parallel to NF-κB and supporting PD-L1 expression, might be destroyed by MALT1 protease activity inhibition. The lack of NF-κB transcription activity reduction might be caused by activation of the signaling adaptor MYD88, which is another prominent NF-κB signaling pathway activator in ABC-DLBCL cells (38, 39). In addition, direct blockade of NF-κB transcription activity by QNZ slightly inhibited the generation of PD-L1^+^ ABC-DLBCL cells in response to Vγ9Vδ2 T lymphocytes, which further indicated that NF-κB is dispensable for the PD-L1^+^ ABC-DLBCL cell generation mediated by MALT1 protease activity.

Recent studies have shown a possible link between metabolic alteration and PD-L1 expression (18, 19). Accordingly, we proposed that MALT1 protease activity might regulate cell metabolic reprogramming to support PD-L1^+^ ABC-DLBCL cell generation. Following this assumption, we observed that MALT1 protease activity inhibition led to low levels of mitochondrial SRC in ABC-DLBCL cells. Mitochondrial SRC has a positive correlation with mitochondrial bioenergetics, which is supported by loading of pyruvate or glutamate into mitochondria (40, 41). In our study, glutamate (m+0) derived from glutamine significantly decreased while glucose-derived pyruvate (m+3) and lactate (m+3) were not influenced by MALT1 protease activity inhibition. This suggested that glutaminolysis might be the target of MALT1 protease activity. Furthermore, importantly, many metabolites (m+0) of the TCA cycle are derived from glutaminolysis. The levels of these metabolites were reduced by MALT1 protease activity inhibition, which was the reason for the increased percentage of glucose-derived metabolites (m+2) in the TCA cycle. Critically, these new observations may help us better understand the metabolic characteristics of ABC-DLBCL cells and provide a basis for developing therapeutic approaches aimed at modulating cell proliferation and apoptosis (42). Because of the function of MALT1 protease activity in glutamate production, we observed that MALT1 protease activity positively regulated GLS1 expression. Therefore, MALT1 protease activity endows ABC-DLBCL cells with high mitochondrial bioenergetics ability, which is mediated by glutaminolysis.

Subsequently, our data show that glutamate derived from glutaminolysis supports mitochondrial bioenergetics and enhances PD-L1 expression in ABC-DLBCL cells in response to Vγ9Vδ2 T lymphocytes. This is distinct from CAD macrophages, in which PD-L1 expression is mechanistically linked to pyruvate-mediated mitochondrial bioenergetics (19). PD-L1 is modulated by the PI3K/AKT and MAPK pathways or several transcription factors, including HIF-1, NF-κB, and STAT3 (14–17). Among these factors, STAT3 is the only essential mediator that was inhibited by glutaminolysis blockade under Vγ9Vδ2 T lymphocyte stress. This suggested that glutamate derived from glutaminolysis plays an essential role in STAT3 activation. At least two possibilities can explain how glutamate activates STAT3. First, glutamate itself may directly activate STAT3 without metabolic processing (43). However, our data argue against this possibility because addition of glutamate did not promote STAT3 activation under TCA cycle blockade. Therefore, the second possibility is more likely: glutamate acted as an intracellular metabolite that entered the TCA cycle for mitochondrial bioenergetics, thereby supporting STAT3 activation. In addition, replacement of glutamate recovered mitochondrial SRC, which further supports this hypothesis.

Glutaminolysis is thought to be the main source of energy production in tumor cells, which show elevated levels of enzymes involved in glutamine/glutamate oxidation compared with those in normal cells (44). This metabolic abnormality maintains a functional TCA cycle for respiration and compensates for increased demands of biosynthetic precursors. In addition, glutaminolysis also plays an important role in regulating redox balance, apoptosis and autophagy in cancer cells. Here, we discovered a novel biological function of glutaminolysis that involves immune evasion, indicating that blocking glutaminolysis might benefit immunotherapy for ABC-DLBCL. GLS is the initial enzyme in glutaminolysis and catalyzes the conversion of glutamine to glutamate, and it is the most extensively studied drug target in the glutaminolysis pathway. In our study, GLS inhibition decreased PD-L1 expression under immune pressure, which attenuated the immunosuppressive property of tumor cells. This suggests that GLS inhibition has the dual functions of inhibiting tumor cell growth and immune evasion. Therefore, characterizing new specific inhibitors for GLS should become a field of intense research.

In summary, as shown in Graphical Abstract, our findings provide evidence that MALT1 protease activity, known to play an important role in NF-κB activation and pathogenesis of ABC-DLBCL, promotes glutaminolysis by up-regulating GLS1 expression and facilitates the generation of PD-L1^+^ ABC-DLBCL cells. The glutaminolysis intermediate glutamate enhances mitochondrial bioenergetics, resulting in STAT3 activation and PD-L1 expression. This endows ABC-DLBCL cells with an immunosuppressive property. Our study provides a new perspective to deepen our understanding of PD-L1^+^ABC-DLBCL cell generation under immune pressure. Hence, manipulation of these pathways could hold enormous potential for the development of effective immunotherapy for ABC-DLBCL.

## Author Contributions

XX and WZ wrote the manuscript, made the figures, and performed research. CG wrote the manuscript. ZF, PL, YX, LiZ, and HZ assisted with the experiments. LeZ revised the manuscript. XX and CS discussed and interpreted the data. YG directed the project, secured funding, and refined the manuscript.

### Conflict of Interest Statement

The authors declare that the research was conducted in the absence of any commercial or financial relationships that could be construed as a potential conflict of interest.
