# Supplementary material for: Glutaminolysis Mediated by MALT1 Protease Activity Facilitates PD-L1 Expression on ABC-DLBCL Cells and Contributes to Their Immune Evasion
Source: Front Oncol. 2018 Dec 18;8:632. doi: 10.3389/fonc.2018.00632 (PMC6305595; doi:10.3389/fonc.2018.00632)
Supplement: Supplementary file 1 [file Data_Sheet_1.doc]

***Supplementary Material***

**Glutaminolysis mediated by MALT1 protease activity facilitates PD-L1 expression on ABC-DLBCL cells and contributes to their immune evasion**

**Xichun Xia, Wei Zhou, Chengbin Guo, Zhen Fu, Leqing Zhu, Peng Li, Yan Xu, Liangyan Zheng, Hua Zhang, Changliang Shan, Yunfei, Gao^#^**

**^#^ Correspondence:** Yunfei Gao (yunfei.gao@utoronto.ca)

**Supplementary Figures**

**
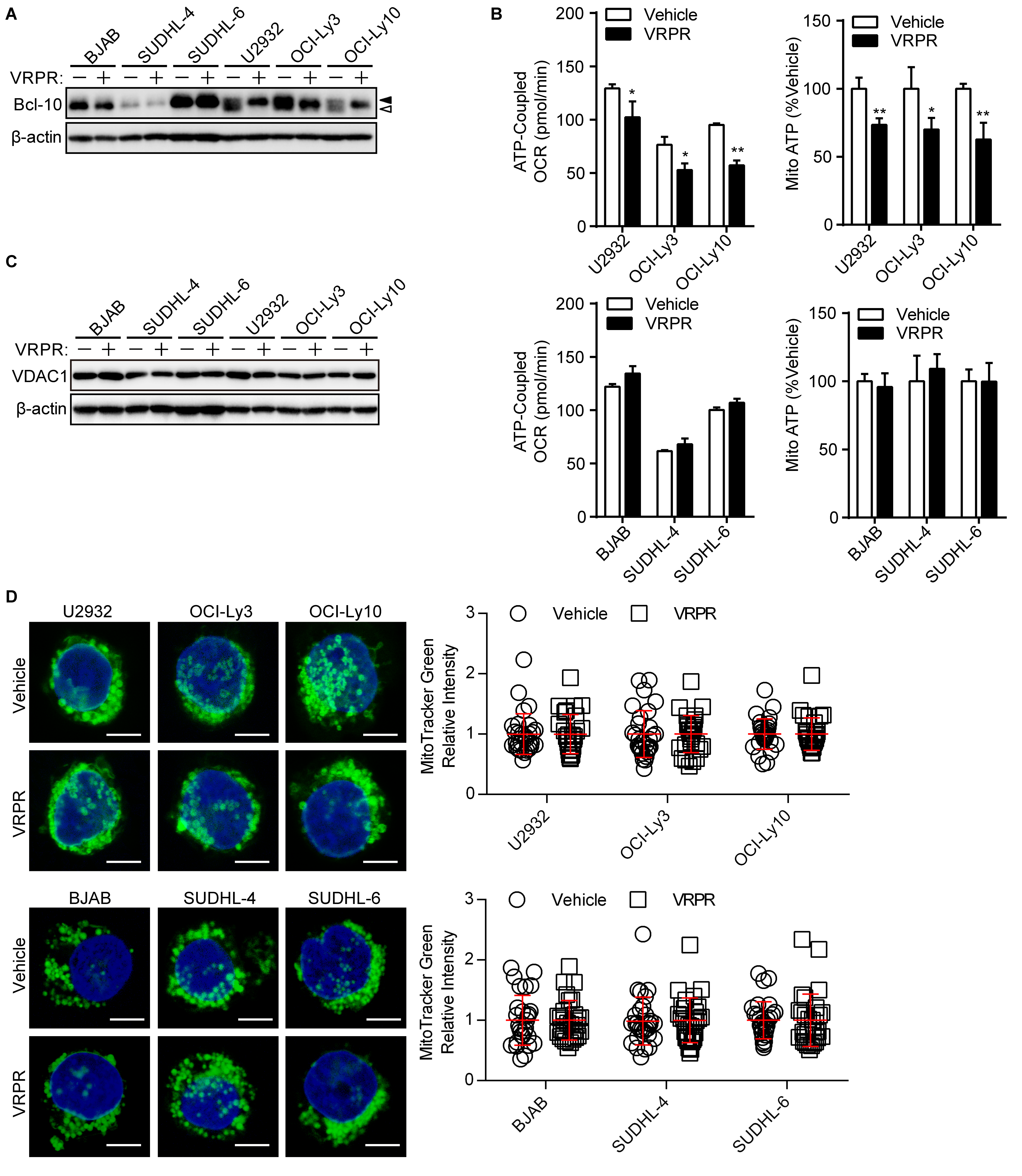
**

**Supplementary Figure 1.** Effects of MALT1 protease activity on mitochondrial biogenesis in DLBCL cells. **(A)** Western blot to detect Bcl-10 cleavage products in DLBCL cells that were treated with VRPR (+) or not (-) for 12 h. Filled arrow, full length Bcl-10; open arrow, Bcl-10 cleavage product. **(B)** ATP-coupled OCR and mitochondrial ATP levels in DLBCL cells that were treated with VRPR or not for 12 h. **(C)** Western blot to detect VDAC in DLBCL cells that were treated with VRPR (+) or not (-) for 12 h. **(D)** Confocal microscopy of DLBCL cells treated with VRPR or not for 12 h, then stained with MitoTracker Green and the DNA-binding dye DAPI. Scale bars, 5 μm. Relative intensity of MitoTracker Green was calculated. Each symbol represents an individual cell. The graphs represent the mean ± SD of three independent experiments. **p* < 0.05; ***p* < 0.01.

**
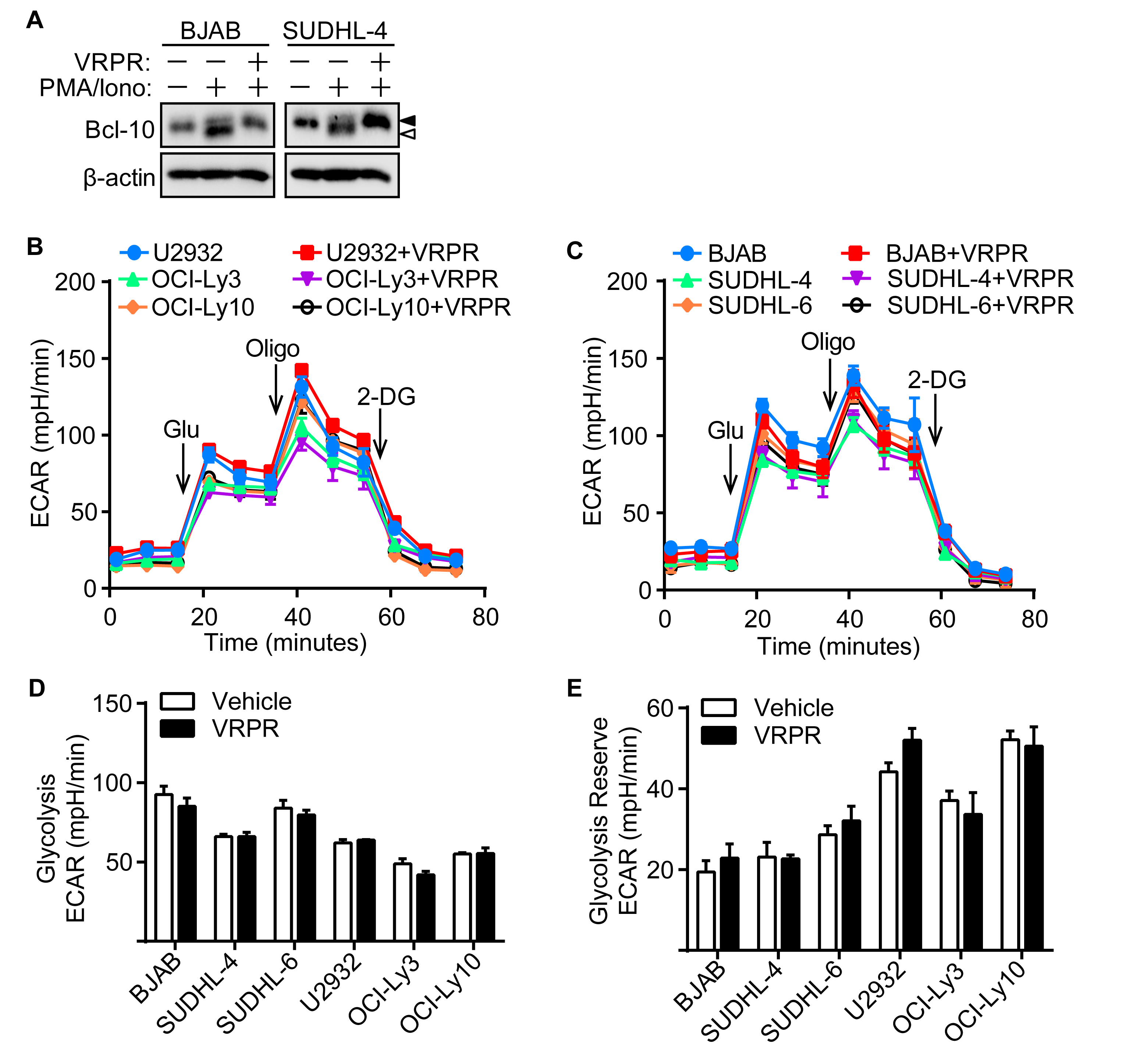
**

**Supplementary Figure 2.** Effects of MALT1 protease activity on glycolytic activity in DLBCL cells. **(A)** Western blot to detect Bcl-10 cleavage products in GCB-DLBCL cells (BJAB and SUDHL-4 cells) that were either left untreated or stimulated for 12 h with PMA/Iono (P/I); zVRPR-fmk was added 12 h before stimulation where indicated. β-Actin was used as the loading control. Glycolysis profiles in ABC-DLBCL cells (**B**) and GCB-DLBCL cells (**C**) treated with VRPR or not for 12 h. ECAR was assayed after consecutive injections of glucose (10 mM), Oligo (1 μM) and 2-DG (50 mM). Glycolysis (**D**) and glycolysis reserve (**E**) were calculated, and the results are shown in the graphs in (**B**) and (**C**), respectively. All the graphs represent as the mean ± SD of three independent experiments.


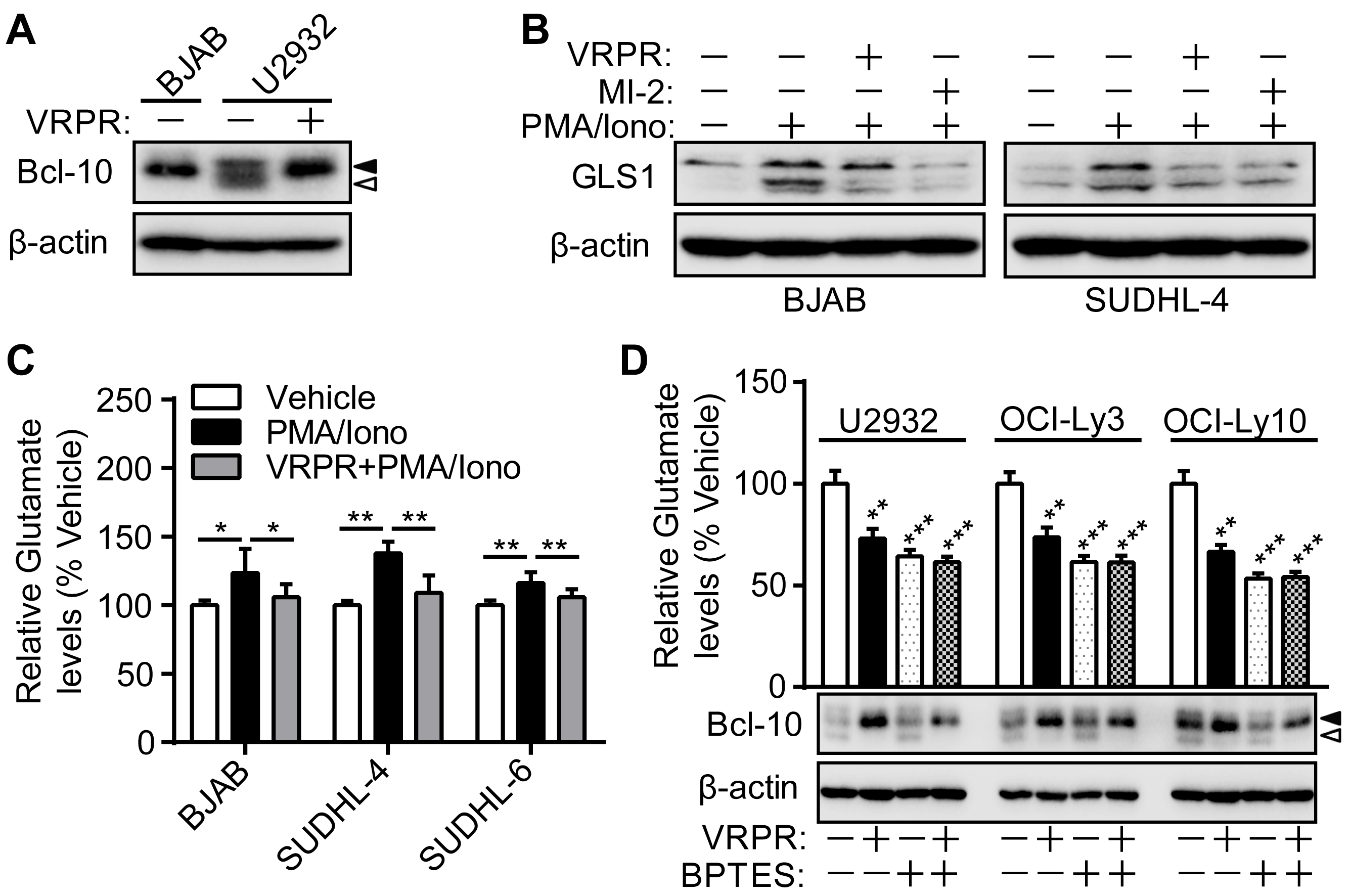


**Supplementary Figure 3.** Correlation between the levels of GLS1 expression and MALT1 protease activity. **(A)** Western blot analysis of the presence of Bcl-10 cleavage products. Lysates from BJAB cells or U2932 cells pretreated with VRPR (+) or not (-) for 12 h were immunoblotted with anti-BCL10 antibody. **(B)** BJAB and SUDHL-4 cell lines were either left untreated or stimulated for 12 h with PMA/Iono; zVRPR-fmk or MI-2 was added 12 h before stimulation where indicated. Western blot to detect GLS1. β-Actin served as the loading control. One representative experiment of three is depicted. **(C)** Relative glutamate levels in GCB-DLBCL cells as in **(B)**. **(D)** Relative glutamate levels and Bcl-10 cleavage products in ABC-DLBCL cells pretreated with vehicle, VRPR, BPTES, or VRPR+BPTES for 12 h. The graphs represent the mean ± SD of three independent experiments. **p* < 0.05; ***p* < 0.01; ****p* < 0.001.


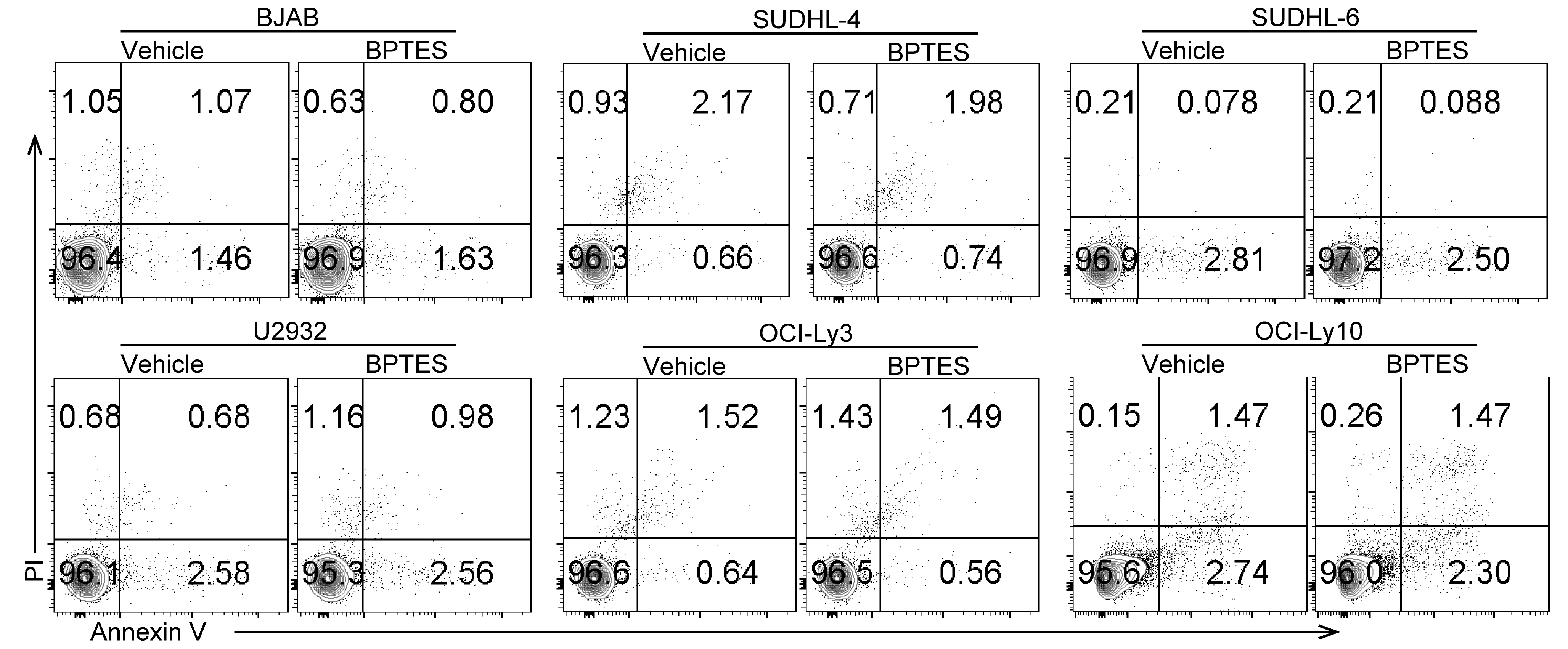


**Supplementary Figure 4.** Effects of BPTES on DLBCL cells survival. Survival of DLBCL cells treated with vehicle or BPTES for 12 h, followed by apoptosis analysis via flow cytometry. The data are representative of three independent experiments.
